# 'Gut health': a new objective in medicine?

**DOI:** 10.1186/1741-7015-9-24

**Published:** 2011-03-14

**Authors:** Stephan C Bischoff

**Affiliations:** 1 Institute of Nutritional Medicine, University of Hohenheim, Fruwirthstr. 12, Stuttgart, D 70599, Germany

## Abstract

'Gut health' is a term increasingly used in the medical literature and by the food industry. It covers multiple positive aspects of the gastrointestinal (GI) tract, such as the effective digestion and absorption of food, the absence of GI illness, normal and stable intestinal microbiota, effective immune status and a state of well-being. From a scientific point of view, however, it is still extremely unclear exactly what gut health is, how it can be defined and how it can be measured. The GI barrier adjacent to the GI microbiota appears to be the key to understanding the complex mechanisms that maintain gut health. Any impairment of the GI barrier can increase the risk of developing infectious, inflammatory and functional GI diseases, as well as extraintestinal diseases such as immune-mediated and metabolic disorders. Less clear, however, is whether GI discomfort in general can also be related to GI barrier functions. In any case, methods of assessing, improving and maintaining gut health-related GI functions are of major interest in preventive medicine.

## Background

In recent times, the term 'gut health' has become increasingly popular, as is evident by its more frequent use in the scientific literature and in the food industry [1]. In contrast to the medical understanding of the Western world, where gut issues are considered rather taboo, gut health is a central theme in Asian medicine, which recognises the abdomen as the location of the soul. "Honoured middle" (onaka) and "centre of the spiritual and physical strength" (hara) are how the Japanese describe our largest organ, the intestine, which for many Europeans is barely more than a simple digestive system which simply has to function [2]. On the other hand, gut health that is more than just a positive gut feeling is now also increasingly recognised in the Western world as a desirable aim and an important physiological condition required for overall good health. There may be two reasons for this. First, a growing number of people do not enjoy good gut health, as is indicated by the high prevalence of functional and organic gastrointestinal (GI) diseases in the Western world. Second, marketing professionals have discovered this concept for their own specific goals [1,3]. However, from a scientific point of view, it is still very unclear what gut health is, how it can be defined and how it can be measured. In the present article, current knowledge of gut health is summarised. Particular emphasis is given to the definition of the term, the underlying mechanisms, how to assess it and how to maintain it. Moreover, the possible impact of gut health for future, prevention-oriented medicine, as well as the need to increase understanding of this condition and to maintain it, is discussed.

## Discussion

### Definition of gut health

The expression 'gut health' lacks clear definition in the scientific literature, although it has been used repeatedly in human medicine [4-7] and in animal health [8,9]. According to the World Health Organisation (WHO) definition of 'health' from 1948, which proposes a positive definition instead of 'the absence of diseases', one might define gut health as a state of physical and mental well-being in the absence of GI complaints that require the consultation of a doctor, in the absence of indications of or risks for bowel disease and in the absence of confirmed bowel disease. Although the WHO defines health as being more than absence of disease, prevention or avoidance of disease is surely part of our understanding of health. Actually, gut health comprises a healthy upper and lower GI tract, although the term might suggest that it is restricted to the lower GI tract. However, other abdominal organs, such as the liver, pancreas, spleen or kidney, are usually not associated with gut health and therefore are not discussed here.

This definition covers the viewpoint of the potentially afflicted individual, who expects a largely symptom-free status that, at the very least, does not require consultation with a physician. It also covers the viewpoint of the doctor, who must bear in mind the potential risks of bowel diseases, particularly malignant bowel disease, even in the absence of any complaints by the patient. This definition, however, is based on exclusions and on more or less subjective criteria [10].

On the basis of the results of discussions within a scientific committee working on gut health issues, five major criteria have been defined that might form the basis for a positive and more objective definition of gut health (Table 1). The criteria could be confirmed in a questionnaire performed in a representative Western population group (SCB, unpublished work).

**Table 1:** Gut health and gastrointestinal health^a^

Five major criteria for a healthy GI system	Specific signs of GI health
Effective digestion and absorption of food	Normal nutritional status and effective absorption of food, water and minerals Regular bowel movement, normal transit time and no abdominal pain Normal stool consistency and rare nausea, vomiting, diarrhoea, constipation and bloating
Absence of GI illness	No acid peptic disease, gastroesophageal reflux disease or other gastric inflammatory disease No enzyme deficiencies or carbohydrate intolerances No IBD, coeliac disease or other inflammatory state No colorectal or other GI cancer
Normal and stable intestinal microbiota	No bacterial overgrowth Normal composition and vitality of the gut microbiome No GI infections or antibiotic-associated diarrhoea
Effective immune status	Effective GI barrier function, normal mucus production and no enhanced bacterial translocation Normal levels of IgA, normal numbers and normal activity of immune cells Immune tolerance and no allergy or mucosal hypersensitivity
Status of well-being	Normal quality of life 'Qi (ch'i)', or positive gut feeling Balanced serotonin production and normal function of the enteric nervous system

^a^GI, gastrointestinal; IBD, inflammatory bowel disease; IgA, immunoglobulin A.

The relevance of gut health is underlined by the fact that the list of intestinal complaints that prompts an individual to consult a doctor is long, and such complaints are very common in the general population. They include symptoms associated with functional dyspepsia and irritable bowel syndrome (IBS) and comprise flatulence, bloating, regurgitation, heartburn, nausea, vomiting, constipation, diarrhoea, food intolerance, incontinence, abdominal pain and cramps, loss of appetite, weight loss and blood in stools. In most cases, such symptoms reflect more or less harmless diseases that might affect quality of life but not mortality. Some of the symptoms, however, such as anorexia, unintended weight loss, dysphagia, continuing vomiting, severe abdominal pain or diarrhoea, melena and hematochezia, have to be interpreted as alarm signals requiring a detailed examination. This is particularly important if such symptoms occur in individuals with a family history of colorectal carcinoma (CRC) or other malignant diseases or in individuals older than 50 years of age with a lack of colonoscopy. In these individuals, malignant GI diseases have to be excluded by appropriate means. Moreover, other chronic GI diseases, such as GI infections, GI immune diseases, inflammatory bowel disease (IBD), and antibiotic-associated diarrhoea, need to be excluded.

### Underlying mechanisms

**Figure 1:**
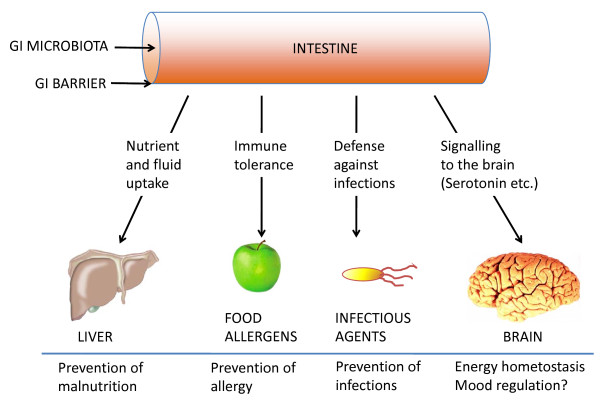
**The intestine's impact on health**. The gastrointestinal tract contributes to health by ensuring digestion and absorption of nutrients, minerals and fluids; by induction of mucosal and systemic tolerance; by defence of the host against infectious and other pathogens; and by signalling from the periphery to the brain. For details and references, see text 'Underlying mechanisms'.

The GI tract contributes to health in many ways. Multiple data clearly indicate that the function of the gut is by far not restricted to food processing and subsequent nutrient and fluid uptake (Figure 1). Animal experiments and some human data have shown that the gut communicates with bacteria that support digestion by their enzymatic capacity [11,12], that the gut regulates major epithelial and immune functions of importance for gut health and health in general [13,14] and that the gut reports to the brain via the N. vagus and hormones about energy uptake and other conditions that might affect mood and general well-being [15]. The details of how the gut has an impact on health in general have been reviewed in detail elsewhere [1,11-15] and are summarised in Figure 1. Of particular interest in this context is the recent finding in germ-free mice that the gut microbiota can directly influence not only GI functions but also the development of behaviour and corresponding neurochemical changes in the brain [16]. The mechanisms of how the gut microbiota contributes to gut health, however, are less clear.

There is now ample evidence that two functional entities are key to achieving and maintaining gut health [17-20]. These entities are the GI microbiome and the GI barrier, which is not just a mechanical barrier assessed by some permeability measurements. Instead, the current understanding of the GI barrier is more complex, since it refers to a functional entity consisting of epithelial defence and metabolic functions, the mucosal immune system and the enteric nervous system (ENS). The importance of this zone in the context of this article is emphasised by the fact that it is the area of sampling and communication between host and luminal content. Therefore, not only permeability tests, which are known to have several serious limitations in themselves [21], but also the whole repertoire of tests recording GI functions need to be considered for the assessment of the GI barrier.

**Figure 2:**
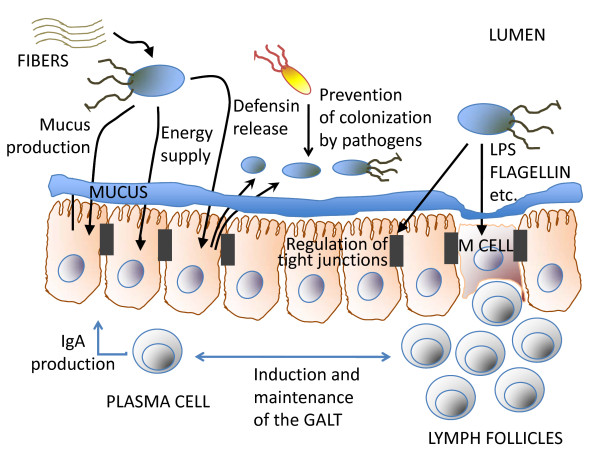
**Mechanisms of action of the intestinal microbiome on the gastrointestinal barrier**. Commensal bacteria support the digestion of fibres and other nutrients, thereby contributing to energy and substrate supply. They regulate epithelial functions such as mucus production in goblet cells, defensin release from Paneth cells and tight junction protein synthesis in normal epithelial cells. They prevent colonisation of pathogens in the gut and regulate the mucosal immune system, for example, by inducing and maintaining gut-associated lymphoid tissue and by stimulating mucosal immunoglobulin A production. For details and references, see text 'Underlying mechanisms'.

The GI microbiome consists of about 10^14 ^bacteria that are mainly located in the large intestine [22,23]. Multiple functions of the GI microbiome have been described (see Figure 2). The GI microbiome prevents colonisation by potentially pathogenic microorganisms, provides energy for the gut wall from undigested food (for example, carbohydrates and other nutrients) and it regulates the mucosal immune system, not only educating the naive infant immune system but also serving as an important source of immune stimulators throughout life [24-29]. Thus, the GI microbiota contributes to energy homeostasis, prevents mucosal infections and likely mitigates immune system hypersensitivity. Most important, it contributes to the maintenance of an intact GI barrier, which seems to be closely related to infectious, inflammatory and allergic diseases [20,30].

Any impairment of the GI microbiome, for example, by administration of oral antibiotics [31-33] or by an unbalanced diet such as a carbohydrate-rich diet [34,35], and SCB, (unpublished work), will affect the functionality of the host's local defence systems. On the other hand, any malfunction of the epithelium, the immune cells or the ENS will affect microbiota diversity and functionality. In particular, the GI barrier, and consequently gut health, will be directly altered not only by local disturbances (such as increased epithelial permeability due to infection or any loss of function of particular immune cells and their mediators) but also by any systemic burden such as reduced oxygenation in intensive care unit patients, malnutrition in cancer patients and the elderly or altered nerve input because of ongoing stress or depression [36-42]. Thus a normal GI microbiota of rich diversity, as well as an intact GI barrier that counteracts the bacteria and cooperates with the commensal flora, is needed to maintain gut health.

The clinical consequences of such interrelationships are only just beginning to be understood. For example, antibiotic effects on gut microbiota composition and functionality can now be assessed by novel GI microbiota analytical technologies. Such innovative methods have been used to show that antibiotics such as ciprofloxacin influence the abundance of about one-third of the bacterial taxa in the gut, decreasing the taxonomic richness, diversity and distribution of the flora. In all the individuals tested in one study [33], the taxonomic composition of the flora closely resembled its pretreatment state four weeks after antibiotic treatment had finished, but there were several taxa that failed to recover within six months. These data conflict with prior assumptions that ciprofloxacin has only a modest effect on the intestinal microbiota. This example illustrates how quickly and unintentionally the GI microbiota can be changed, and we are just starting to realise the possible long-term consequences of such manipulations. Further establishment of diagnostic means to assess quickly and accurately the GI microbiome composition using molecular techniques will in the future allow us not only to describe in more detail the multiple relationships between the colonic microbiota and poor gut health but also to assess the impact of interventions intended to restore a healthy microbiota in the gut, such as dietary changes and the administration of probiotics, prebiotics or antibiotics [30,43,44].

The mucosal immune system of the GI tract both controls the GI microbiome and depends on it. The permanent challenge of bacterial antigens to the mucosal immune system is required for its normal development and function [24,25]. In this context, it is not surprising that the GI immune system contains cells capable of recognising bacterial antigens by specific receptors, such as T-cell receptors (TCRs) and B cell-derived, surface-bound antibodies of the adaptive immune system, as well as Toll-like receptors (TLRs) and other pattern recognition receptors (PRRs) of the innate immune system. Dendritic cells (via TLRs), lymphocytes (via TCRs and antibodies) and innate immune cells such as macrophages and mast cells (via TLRs and other PRRs) are also involved in communication between the GI microbiome and the GI immune system so that any danger from pathogens can be recognised and also to maintain the friendly coexistence of bacteria and host in the gut [45-48].

To achieve the defence of the host against luminal bacteria and other potentially harmful substances, the GI immune system is equipped with specific tools, such as the plasma cell-dependent immunoglobulin A (IgA) defence system, goblet cell-derived mucus production and the synthesis of antimicrobial peptides such as defensins by Paneth cells [20,49-51]. All of these tools play a part in controlling the GI microbiome and protecting the host against invasion of luminal bacteria through the gut wall. Under normal conditions, these mechanisms also prevent direct contact between commensal bacteria and the GI epithelium [52]. Moreover, the GI immune system allows regulation of inflammatory responses to harmless antigens, such as food antigens or bacterial antigens derived from commensals, by mechanisms that together result in mucosal tolerance. The details of these mechanisms are not fully understood, but it is clear from numerous observations that loss of bacterial challenge and loss of immune tolerance results in severe hypersensitivity reactions, leading to chronic inflammatory states such as allergic disease, autoimmune disease and IBD [53,54]. Thus the GI immune system contributes to both the defence against and the acceptance of bacteria, and it fends off bacteria yet also needs them, all of which illustrate the complex balance of interactions between the GI microbiome and the GI immune system that protect the host and maintain gut health.

In addition to the mucosal immune system, the ENS is another perplexing and complex control and defence system that is starting to become understood. The ENS contains 10^8 ^neurons and forms the largest neuronal network outside the brain. It monitors luminal conditions via sensory receptors and primary afferent neurons activated by secretagogues from enterochromaffin cells or mast cells such as biogenic amines (serotonin and histamine) or proteases [55,56]. The ENS is strikingly independent from the central nervous system (CNS), yet it regulates almost all major functions of the gut, such as epithelial secretion, absorption and permeability; immune functions; and, as shown most recently, even the GI microbiota [40,41]. GI infections, oral administration of antibiotics and chronic diseases such as IBS are clearly associated with morphological and functional changes in the ENS, which emphasises its role in mucosal defence. The ENS mediates multiple as yet undefined signals to the brain that seem to touch our awareness only under pathological conditions. Recent experimental studies strongly suggest that luminal conditions and signals, as well as the intestinal microbiota, are integrated into a gut-brain axis (GBA). For example, the stress-induced adrenocorticotropin hormone response in animals is much more pronounced in germ-free mice than in colonised animals [57]. Such interrelationships might provide a scientific basis for any 'gut feeling' or the above-mentioned Asian understanding of the gut being the centre of spiritual and physical strength. The first hints that CNS diseases such as hepatic encephalopathy, depression and autism spectrum disorder might be treated by modulating the GI microbiome (for example, with the prebiotic and laxative lactulose, sugar-reduced diets or antibiotics) is a further argument for the relevance of the GBA [58-60]. Moreover, experimental data have shown that probiotics, by modifying the GI microbiome, can affect both the ENS [61] and the CNS [62], which highlights the relevance of the CNS for gut health. Such findings might explain, at least in part, why probiotics can show effects outside the GI tract.

The question of genetic factors that might influence gut health is difficult to answer, because valid data are lacking. Although a few IBS studies on familial associations and gene polymorphisms have suggested that genetic factors might play a role in the pathogenesis of this disease [63,64], the converse argument that genetic factors determine gut health is not justified yet. In particular, polymorphisms have been examined only in rather small groups, and familial associations do not exclude that environmental factors, including biological, psychological and sociological components, also play a role. Moreover, apart from human gene polymorphisms, bacterial genetic variations have to be considered in this context [65]. Genetic and environmental factors affecting gut health are not mutually exclusive, since most chronic diseases, including somatic syndromes and IBS, are likely related to both [63,66]. In this context, it is interesting to note that IBS is now also recognised from an epigenetic perspective on the basis of animal studies and a few human data (reviewed in [67]). This emerging field could in the future improve our understanding of how diet and the gut microbiota might influence gut health. Most excitingly, recent data in germ-free and colonised mice indicate that commensal microbiota profoundly shape the invariant natural killer T-cell compartment, a major component of the host immune system, through an epigenetic mechanism [68]. Other groups have shown that selected bacterial strains can induce regulatory cells that protect against pathogen-triggered or allergen-induced inflammation [69]. Such data strongly suggest that both genetic and epigenetic factors are involved in the maintenance of gut health.

In summary, the key to understanding gut health is an awareness that the GI barrier consists of multiple epithelial functions, the mucosal immune system, the ENS as well as the tissue matrix, the muscle layers and the blood supply. The GI barrier not only protects the host against potential dangers from the GI lumen but also allows food and liquid uptake, beneficial cross-talk to commensal bacteria and immune tolerance against harmless antigens. An intact GI barrier maintains gut health, whereas disturbance of GI barrier functions is increasingly recognised as an early but essential step in the pathogenesis of many GI diseases and even extraintestinal diseases.

### The GI microbiota and the GI barrier: implications for disease

There is an ever-growing list of diseases for which alterations of the GI barrier have emerged as a crucial event in disease pathogenesis, and this list includes relevant GI and extraintestinal diseases (Table 2). It is striking that many of these diseases are characterised by an altered GI microbiota, further suggesting a link between GI barrier function and GI microbiota composition. The references cited in Table 2 demonstrate several examples of an association between changes in microbiota and disease, although the mechanisms of interaction are not always evident. On the other hand, because of such associations, it is tempting to speculate that the maintenance of normal microbiota and a stable GI barrier contributes to gut health and likely to health in general.

**Table 2:** Diseases thought to be associated with GI barrier and GI microbiota^a^

Location	Diseases for which the GI barrier plays a central role in pathogenesis	Diseases associated with an altered composition or function of the GI microbiota
Intestinal	Infectious diarrhoea [26,36] Inflammatory bowel disease [130-132] Coeliac disease [131] Irritable bowel syndrome [133]	Inflammatory bowel disease [138,139] Coeliac disease [140] Irritable bowel syndrome [141,142]
Extraintestinal	Allergic diseases [20,134,135] Autoimmune diseases and arthritis [17] Obesity, fatty liver disease and nonalcoholic steatohepatitis (NASH) [35,136,137] Systemic inflammatory response syndrome (SIRS) and sepsis in ICU patients [36,37] Malnutrition [40]	Allergic diseases [143,144] Arthritis [145] Obesity [125]

^a^GI, gastrointestinal; ICU, intensive care unit.

### How to test gut health?

Gut health is frequently talked and written about but rarely if ever measured, because the boundaries and characteristics of this kind of 'wellness' are ill-defined. Diagnostic efforts are mostly oriented towards measurement of pathological situations, but the progress and importance of preventive medicine makes assessment of normal organ functions an increasingly relevant exercise. To assess gut health, diagnostic methods must cover both subjective complaints and objective parameters.

Complaints cannot be assessed without registering an individual's history by using a questionnaire or performing a structured personal dialogue, for example, with the doctor. Questionnaires, ideally combined with some biomarkers of prognostic relevance, are also appropriate to screen a population and to accustom people to this particular issue, which is still often considered taboo. Such an approach requires validated tools adapted to bowel-related complaints and symptoms that would enable clinicians to record improvements in well-being, quality of life and prognosis in selected populations (Table 3).

**Table 3:** Assessment of normal gut function^a^

Assessments and parameters		Descriptions
Subjective assessments of well-being		
Validated questionnaires useful to assess quality of life and gut health	IBS-Quality of Life (IBS-QOL)	Validated for assessment of quality of life specific to IBS: 34 questions
	Inflammatory Bowel Disease Questionnaire (IBDQ)	Validated for assessment of health-related quality of life (HRQoL) in adult patients with IBD
	Bowel Disease Questionnaire (BDQ)	Validated to distinguish patients with functional and organic GI disease
	Health Status Questionnaire (HSQ-12)	Validated for assessment of HRQoL in the general population
	Short Form Health Survey SF-12 (SF-12)	Validated for assessment of HRQoL in the general population
GI symptom scores	IBS Severity Scoring System (IBS-SSS)	Validated for scoring lower GI symptoms on the basis of nine questions; range of 0 to 500 points
	Short Form Leeds Dyspepsia Questionnaire (SF-LDQ)	Validated instrument for measuring the presence and severity of dyspepsia
	Gastrointestinal Symptom (GIS) profile	Validated for assessment of symptoms of functional dyspepsia; 10 questions
	Subject's Global Assessment of Relief (SGA)	Validated assessment of the impact of treatment on IBS-related symptoms; 1 question
	IBS Global Assessment of Improvement (IBS-GAI) ("adequate relief")	Asks participants if, compared to the way they felt before entering the study, their IBS symptoms have changed over the past 7 days; 1 question
	Functional Bowel Disorder Severity Index (FBDSI)	Validated score for assessment of patient perception of abdominal pain in IBS
	Numeric Rating Scale (NRS) for assessment of pain	Validated in IBS patients
	Visceral Sensitivity Index (VSI)	Validated psychometric instrument that measures GI symptom-specific anxiety
	Bristol Stool Scale/Bristol Stool Chart	A medical aid designed to classify the form of human faeces into seven categories
	Gastrointestinal Symptom Rating Scale	
Eating habits	Food frequency questionnaire	7-day diet history assessed using computer software
Objective parameters		
Markers of functionality	Gastric function	pH metry in the esophagus [6,7] and stomach [1-3], gastroesophageal reflux episodes (< 50/day, <60 minutes total), viscosity of the luminal content using a viscometer, stool weight (> 100 g/d, <500 g/d) and stool consistency (water content)
	Permeability measurements	Tracer molecules (lactulose/mannitol, ^51^Cr-EDTA, PEG);
	Motility tests	Barostat, gastric scintigraphy, ^13^C urea breath test (gastric emptying) and lactulose hydrogen breath test (normal range: 40 to 240 hours)
	Transit time	Radiopaque pellets (Hinton test) and isotope-labelled test meal (normal range: 24 to 168 hours)
	Digestion parameters	Stool elastase (> 200/g) and stool fat (< 7 g/d), carbohydrate breath tests, anthropometry and micronutrient analysis
Markers of intestinal integrity	Epithelial integrity	Histology (villus height/crypt depth ratio, mitosis and apoptosis), mucus secretion (mucins and trefoil peptides) and Ussing chamber (ion fluxes and electric potentials)
	Specific molecules	E-cadherin, growth factors, tight junction molecules, α_1_-antitrypsin in faeces and LPS in blood
	Antimicrobial peptides	α- and β-defensins, calprotectin, lysozyme or neutrophil-derived elastase in faeces
Marker of intact immunity	Cell counts and phenotyping	Differential blood count and FACS analysis, histopathology of intestinal biopsies and immunohistochemistry of intestinal biopsies
	Cell mediators and cytokines	Inflammatory cytokines (IL-1, IL-6 and TNFα), anti-inflammatory cytokines (IL-10 and TGFβ), regulatory cytokines (IL-2, sIL-2R, IL-4, IL-5 and so on), proteases (tryptase, chymases, chymotrypsin and so on), immunoglobulins (IgA, sIgA and IgE) and others (retinoic acid, neuropeptides and so on)
	Functional assays	Cell cultures and cocultures, DTH response, phagocytosis, chemotaxis, oxidative burst (superoxide anion generation) and NK cell activity
Analysis of the intestinal microbiome	Classical approaches	Bacterial culture and toxin measurements
	New approaches	Metagenomics (PCR and full bacterial sequencing), metabonomics (metabolic capacity of the microbiome)

^a^IBS, irritable bowel syndrome; IBD, inflammatory bowel disease; GI, gastrointestinal; ^51^CR-EDTA, chromium ethylenediaminetetraacetic acid; PEG, polyethylene glycol; LPS, lipopolysaccharide; FACS, fluorescence-activated cell sorting; IL, interleukin; TNF, tumour necrosis factor; TGF, transforming growth factor; sIL-2R, soluble interleukin 2 receptor; Ig, immunoglobulin; sIgA, secretory immunoglobulin A; DTH, delayed-type hypersensitivity; NK, natural killer; PCR, polymerase chain reaction.

One such questionnaire is the Inflammatory Bowel Disease Questionnaire (IBDQ), a validated and reliable tool used to measure health-related quality of life (HRQoL) in adult patients with IBD, ulcerative colitis and Crohn's disease [70]. Whether the IBDQ can also be used to assess HRQoL in the general population still needs to be tested. One advantage of this questionnaire is that it has been adapted and validated in several languages and cultural milieus. A more general tool is the Bowel Disease Questionnaire (BDQ), which aims to distinguish patients with functional GI disease from those with other conditions such as IBD or CRC [71]. Logistic regression and discriminant analyses have shown that the BDQ is a valid measure of symptoms of functional GI disease. Probably the most suitable HRQoL assessment tool is the Irritable Bowel Syndrome-Quality of Life questionnaire, because IBS is the disease entity closest to the borderline between gut health and GI disease [72]. The Health Status Questionnaire-12 (HSQ-12) is another reliable, valid, low-cost measure of health status that was created to assess the general population [73]. The HSQ-12, in contrast to the also validated 12-item Short Form Health Survey (SF-12), can distinguish between people with and without dementia [74]. On the other hand, both the HSQ-12 and the SF-12 assess general health status, but not specifically gut health. Therefore, a short general questionnaire could be combined with a gut-related one, although this combination has yet to be evaluated.

It may be helpful to combine such questionnaires with, for example, the Gastrointestinal Symptom Rating Scale, consisting of 15 questions on symptoms [75], or the Bristol Stool Scale, which classifies the form of human faeces into seven categories, with types 1 and 2 indicating constipation, types 3 and 4 being the 'ideal stools', and types 5 to 7 suggesting diarrhoea or bowel urgency [76]. Other disease-related GI symptom scales that have been validated are the IBS Severity Scoring System for scoring lower GI tract symptoms and the Leeds Dyspepsia Questionnaire or the Gastrointestinal Symptom profile for scoring upper GI tract symptoms [77-79]. Moreover, scores have been developed and tested which assess particular symptoms, such as pain, using the Functional Bowel Disorder Severity Index or the Numeric Rating Scale [80,81], as well as scores that assess anxiety using the Visceral Sensitivity Index [82]. In clinical trials, overall improvement of symptoms has been assessed by using singular global questions such as the IBS Global Assessment of Improvement or the Subject's Global Assessment of Relief, which have yielded reliable results [83,84].

Bowel functions are extremely complex and variable; therefore, objective assessment is a difficult task. Nevertheless, multiple approaches have been used to assess a range of bowel functions. For example, glucose challenge with a subsequent hydrogen breath test was believed to identify individuals with so-called 'abnormal bacterial colonisation' of the intestine as a possible cause of deterioration of gut health. Over the past few years, there have been impressive developments in techniques for analysing the human microbiome, such as using metagenomic-metabonomic linkage analyses or small subunit ribosomal RNA hypervariable tag sequencing [7,30,43,44,85]. Microbiome analysis, however, is still considered inappropriate for routine diagnosis of gut function or gut health. However, even modern molecular techniques do not yet allow the definition of what can be considered a 'normal' or 'optimal' microbiome composition. On the other hand, an increasing number of immunological parameters have become available, among which cell counts and cell phenotyping by flow cytometry and immunohistology and quantification of cytokines, antibodies and mediators are the most well-known tools (Table 3).

Despite an increasing number of more or less validated laboratory methods for objective assessment of bowel function, the perception of the individual should never be ignored. Only direct conversation between a doctor and patient allows the opportunity of being able to register GI-specific sensations that are pleasant, which are primarily related to the intake of meals and the evacuation of faeces, that is, gratifying sensations, such as satiation and complete rectal evacuation. Other physiological events, such as eructation and the emission of wind from the anus, may also contribute to GI well-being. Sensations related to thirst, taste, smell and the desire of specific types of foods (salts or sweets, for example) should also be considered part of this concept. Patients with so-called functional GI diseases lose such pleasant sensations, the perception of which will be modulated by age, sex, cultural background and psychological stress.

### How to maintain gut health?

Our knowledge about how to maintain or restore gut health is limited in evidence-based medicine terms, but general observations suggest that there is a wide range of possible ways to support gut health and GI well-being. Current medical research is much more focused on the treatment of defined GI disease rather than on the secondary or even primary prevention of disease. For example, we know of several effective drugs to treat acute IBD and a few to support remission, but almost nothing to prevent IBD, a situation that might be related not only to pathophysiological circumstances. On the other hand, preventive medicine is increasingly perceived as being important in medical and economic terms, particularly in the field of gastroenterology, where we have to deal with a broad grey area between suboptimal health and disease.

Many approaches to maintaining gut health and preventing GI diseases such as infection, antibiotic-associated diarrhoea, IBD, IBS, food allergy and so on are related to the hygiene hypothesis. This concept maintains that any disturbance of the balance between the microbiome and the mucosal immune system will lead to impairment of the GI barrier and subsequently to an increased risk to gut health and subsequent development of GI disease [53,86-88]. Therefore, any conditions that might disturb the intestinal microbiome and the mucosal immune system should be avoided, such as not only unbalanced diet and lack of exercise but also extreme exercise and any type of chronic stress. In recent publications, high-fat as well as high-fructose diets have been shown to disturb the GI barrier and, in this way, to induce fatty liver disease and subclinical inflammatory conditions associated with metabolic disturbances [35,89,90]. On the other hand, dietary changes have been shown to help prevent major diseases such as allergy, obesity and cancer [20,90,91]. Therefore, a balanced diet that includes high vegetable and fibre content and moderate consumption of red meat to prevent colon cancer [92,93], or an individualized elimination diet in selected individuals with food intolerances, food allergy or coeliac disease [94,95], might contribute to gut health. Moreover, tobacco abstinence, moderate alcohol consumption, maintenance of normal body weight, avoidance of nonsteroidal anti-inflammatory drug (NSAID) ingestion and control of stress can support gut health. Systematic strategies to improve lifestyle and to avoid or reduce stress that have been validated in controlled trials are rare; however, meditative methods that often originate from traditional Chinese medicine and other Asiatic cultures (for example, ayurveda and tai-chi) are enjoying growing popularity and becoming increasingly accepted by health professionals as valuable tools to maintain gut health and general well-being [96,97].

Chemoprevention by taking aspirin, cyclooxygenase 2 inhibitors and calcium may reduce the recurrence of adenomas and/or the incidence of advanced adenomas in individuals with an increased risk of CRC, and taking aspirin may reduce the incidence of CRC in the general population [98]. However, both aspirin and NSAIDs are associated with adverse effects, so it will be important to consider the risk-benefit ratio before recommending these agents for chemoprevention. Other chemopreventive strategies, such as retinoid-based therapy and tumour necrosis factor-related, apoptosis-inducing ligand in patients at risk for CRC, are based on animal experiments and need to be confirmed in human studies [99]. In the upper GI tract, chemopreventive strategies, such as the use of celcoxib, have been shown to be less successful [100]; therefore, endoscopic surveillance strategies are still indispensable in patients at risk for GI malignancies.

An interesting idea is whether gut health can be further supported by using modulators of the intestinal microbiome or the GI barrier, such as probiotics or prebiotics. Indeed, it has been shown that chronic bowel diseases such as IBD are associated with adherence of commensal bacteria to the otherwise sterile intestinal epithelium [101] and that selected probiotics can prevent the adhesion of pathogenic bacteria to the intestinal mucosa [102] or restore leaky gut by improving the molecular composition of tight junctions [103,104]. Moreover, probiotic bacteria can support the normal development of the mucosal immune system, such as through the C-C chemokine receptor 6 (*CCR6*) gene expressed on lamina propria lymphocytes [24] and the production of protective IgA and antimicrobial defensins such as the CCR6 ligand human β-defensin 2 (or hBD2) [105,106] that are both lacking in many chronic IBDs [51]. These few selected examples indicate the strong rationale for using probiotics, possibly also in synergistic combinations with prebiotics, to maintain gut health.

We have good data now, even meta-analyses including more than 1,000 individuals, on probiotic effects in preventing or attenuating acute gastroenteritis, antibiotic-associated diarrhoea, IBS and chronic constipation and necrotising enterocolitis in small infants (reviewed in [30]). Apart from this therapeutic approach, it is still difficult to give a general recommendation of probiotics as a preventive measure. The number of prospective, controlled human trials that have been conducted in this field is rather small at present. Limited knowledge within the scientific community and regulatory authorities on suitable markers, as well as regarding the details of how to perform such trials, is the main reason for the lack of appropriate long-term, cost-intensive prevention trials. Recently, a few trials have yielded promising but rather preliminary clinical data or surrogate marker analysis [107-109]. The challenge for the future, based on our growing expertise, is to select adequate measurable parameters, to consider interindividual variations in the composition of the intestinal microbiome and to exclude numerous other putative confounding factors, since regulatory authorities require data from human trials, not just for drugs but also for dietary supplements, to provide scientific support for health claims [1].

### Outlook for gut health: a new objective in medicine?

Medicine in the Western world is undergoing substantial changes. The more advances there are in treatment options, the less affordable they become for everybody. Breaking free of this vicious cycle requires effort not only to manage advanced disease states but also to intervene at an early stage. Only effective strategies in preventive medicine keep our health systems affordable; therefore, we must draw more attention to the maintenance of health, and this includes gut health [110-113]. One of the major areas that needs to be addressed is the gut, because epidemiologic and economic data point to the relevance of diseases such as IBS, IBD and CRC, which are, in principle, to a large extent avoidable. So far, great effort has been made to improve CRC prevention strategies, with variable success [114,115]. The success rate is limited less by the accuracy of the diagnostic tools and more by dependence on the behaviour of a population and the acceptance of screening programs. Therefore, a major effort in the future must be made to improve acceptance rates of guidelines by physicians and of screenings by the afflicted populations [116]. Moreover, to improve gut health, efforts must not be restricted to malignant diseases such as CRC but must also include inflammatory and so-called functional GI diseases such as IBD and IBS.

Consideration of the overall burden and economic impact of IBS, which afflicts perhaps 10% of the population in a moderate to severe way, and almost everyone from time to time at lower degrees, makes it clear that every improvement in understanding IBS and care of IBS is a positive step forward for gut health maintenance [117]. There is now overwhelming evidence that the intestinal microbiome is not only changed but also involved in the pathogenesis of IBS and stress-induced GI dysfunction [118,119]. Probiotic intervention, however, still needs to be improved [120,121]. We speak about 'good health', and the bacteria that live in our gut are said to be 'in balance' when the number of 'good bacteria' outnumber the number of 'bad bacteria'; however, we need to learn more about the mechanisms underlying such expressions. Moreover, the mechanism of activity for probiotics inside and beyond the gut is also far from clear [122,123]. Overcoming these deficiencies requires a new focus in research and the overcoming of taboos that still exist for gut issues, which are regarded as 'ugly' and 'dirty'. The most relevant first step would be to declare the maintenance of gut health and the improvement of preventive medicine as major goals in future medicine, which would have a positive effect on people's awareness, on research and education and on health insurance coverage.

To focus on new gut health parameters such as microbiome gene analysis, and to combine them with tools such as genomics and metabolomics, might become the basis for a new kind of personalised medicine that nutritionists and dieticians have been seeking for several years [4-6,85]. Indeed, recent genome analysis of the host has revealed that the success of dietetic recommendations probably depends on the genetic makeup of the individual [124]. Similar findings have been described by microbiologists who found that obesity risk and treatment response are associated with the particular composition of the intestinal microbiome [125,126]. Possibly, gut health and general health are dependent not only on the genetics of the host but also on the genetics of the commensal bacteria. The tools required to answer such questions are rapidly advancing and have already allowed adequate analysis in research settings and perhaps also in the near future in health care settings [1,27]. The use of such tools in daily practice for diagnostic purposes and for new drug development [127], together with improved communication about gut issues as proposed by several national initiatives in the United Kingdom [128], the Netherlands [129] and Germany (initiative of the Felix-Burda foundation; see http://www.darmgesundheits-check.de/), would likely improve both our understanding of gut health and our approaches to maintaining it. By doing so, an exciting new contribution to preventive, therapeutic and more economically efficient medical practice could be offered.

## Summary

The term 'gut health' has become popular; however, from a scientific point of view, the term is poorly defined and used in different contexts. It covers all aspects, ranging from the Asian understanding of the gut as the middle of spiritual and physical strength to the Western understanding of the GI barrier as a central body site interacting with the environment and involved in the pathophysiology of many intestinal and extraintestinal diseases. The expression 'gut health' lacks clear definition in the scientific literature, although it has been used repeatedly in human medicine and in animal health. Herein a positive definition of 'gut health' is proposed in accordance with the WHO definition of health: 'Gut health is a state of physical and mental well-being in the absence of gastrointestinal complaints that require the consultation of a doctor, in the absence of indications or risks of bowel disease, and in the absence of confirmed bowel disease' (source: Constitution of the World Helath Organization, New York 1946, see http://www.who.int/governance/eb/who_constitution_en.pdf).

The relevance of gut health is underlined by the fact that its loss is characterised by a large variety of symptoms, often prompting an individual to consult a doctor. Such symptoms are associated with the widespread disease IBS and comprise flatulence, bloating, regurgitation, heartburn, nausea, vomiting, constipation, diarrhoea, food intolerance, incontinence, abdominal pain and cramps, loss of appetite, weight loss and blood in stools. Functional oesophageal and gastric diseases must also be considered in this context. In most cases, such symptoms reflect more or less harmless diseases that might affect quality of life but not mortality. Some of the symptoms, however, such as anorexia, unintended weight loss, dysphagia, continuing vomiting, severe abdominal pain or diarrhoea, melena and hematochezia, must be interpreted as alarm signals requiring a detailed examination. This is particularly important if such symptoms occur in individuals with a family history of CRC or in older adults who have never had a colonoscopy.

The mechanisms of ensuring gut health are complex and comprise a healthy lifestyle, a balanced diet, normal GI perfusion, normal GI microbiome and likely a stable mental status. Any major impairment of these mechanisms leads to a breakdown of the GI barrier that defends us against environmental and endogenous hazards. Of particular interest in this context is a better understanding of the interaction between the GI microbiome and the mucosal immune system, which both depend on each other to form an intact GI barrier. Moreover, multiple epithelial functions, apart from nutrient absorption, and functions of the enteric nervous system seem to be of crucial importance to maintaining a functional GI barrier and ultimately gut health. The measurement of gut health is not well established. Two approaches have been recognised: assessment of subjective complaints by using validated questionnaires indicating gut health and assessment of physiological GI function by defining and measuring validated biomarkers related to gut health. Both approaches are equivalent and cannot replace each other.

Gut health can offer a new approach to preventive medicine if we learn more about how to achieve and maintain it. Current medical research is much more focused on the treatment of defined GI diseases rather than on the secondary or even primary prevention of disease. However, preventive medicine is increasingly perceived as being important in medical and economic terms, particularly in the field of gastroenterology. Therefore, scientifically justified approaches to maintaining gut health and to preventing GI diseases are welcome. Although this is an area with many open questions, we have started to learn that lifestyle characteristics, such as balanced diet, moderate but regular exercise and avoidance of chronic stress, but also defined products such as select pre- and probiotics, can support gut health. This topic will concern us even more in the future if we succeed at increasing our knowledge of the underlying mechanisms and how to influence them in a positive way.

## Competing interests

The author declares that they have no competing interests.

## Author's information

SCB has been Professor of Medicine and Chair of Nutritional Medicine and Prevention at the University of Hohenheim, Stuttgart, Germany, since 2004. He is trained as a gastroenterologist and allergologist and used to work in Mainz and Hanover, Germany; Strasburg, France; Bern, Switzerland; and at Columbia University, New York, NY, USA. In 2005, he became Vice Dean of the Faculty of Life Sciences and member of the managing board of the Life Science Centre, Hohenheim, Germany. Since 2008, he has been an elected officer and board member of the European Society of Parenteral and Enteral Nutrition; since 2009, he has been Medical Director of the Centre of Nutritional Medicine of the Universities of Hohenheim and Tübingen, Germany, and Chair of the Scientific Council of the Max-Rubner-Institute, Karlsruhe, Germany. In 2010, he became President-elect of the German Society of Clinical Nutrition, and he founded the new German Society of Mucosal Immunology and the Microbiome. He is the author of more than 100 scientific papers, author and editor of major textbooks and a member of several editorial boards of scientific journals.

## Pre-publication history

The pre-publication history for this paper can be accessed here:

http://www.biomedcentral.com/1741-7015/9/24/prepub
